# A Novel EGFR Targeted Immunotoxin Based on Cetuximab and Type 1 RIP Quinoin Overcomes the Cetuximab Resistance in Colorectal Cancer Cells

**DOI:** 10.3390/toxins15010057

**Published:** 2023-01-09

**Authors:** Nicola Landi, Vincenza Ciaramella, Sara Ragucci, Angela Chambery, Fortunato Ciardiello, Paolo V. Pedone, Teresa Troiani, Antimo Di Maro

**Affiliations:** 1 Department of Environmental, Biological and Pharmaceutical Sciences and Technologies (DiSTABiF), University of Campania ‘Luigi Vanvitelli’, Via Vivaldi 43, 81100 Caserta, Italy; 2 Medical Oncology, Department of Precision Medicine, University of Campania ‘Luigi Vanvitelli’, Via S. Pansini 5, 80131 Napoli, Italy

**Keywords:** *Chenopodium quinoa*, chemical conjugation, cytotoxicity, GEO-CR cells, monoclonal antibody

## Abstract

Cetuximab is a monoclonal antibody blocking the epidermal growth factor receptor (EGFR) in metastatic colorectal cancer (mCRC). However, cetuximab treatment has no clinical benefits in patients affected by mCRC with KRAS mutation or in the presence of constitutive activation of signalling pathways acting downstream of the EGFR. The aim of this study was to improve cetuximab’s therapeutic action by conjugating cetuximab with the type 1 ribosome inactivating protein (RIP) quinoin isolated from quinoa seeds. A chemical conjugation strategy based on the use of heterobifunctional reagent succinimidyl 3-(2-pyridyldithio)propionate (SPDP) was applied to obtain the antibody-type 1 RIP chimeric immunoconjugate. The immunotoxin was then purified by chromatographic technique, and its enzymatic action was evaluated compared to quinoin alone. Functional assays were performed to test the cytotoxic action of the quinoin cetuximab immunoconjugate against the cetuximab-resistant GEO-CR cells. The novel quinoin cetuximab immunoconjugate showed a significant dose-dependent cytotoxicity towards GEO-CR cells, achieving IC_50_ values of 27.7 nM (~5.0 μg/mL) at 72 h compared to cetuximab (IC_50_ = 176.7 nM) or quinoin (IC_50_ = 149.3 nM) alone assayed in equimolar amounts. These results support the therapeutic potential of quinoin cetuximab immunoconjugate for the EGFR targeted therapy, providing a promising candidate for further development towards clinical use in the treatment of cetuximab-resistant metastatic colorectal cancer.

## 1. Introduction

Immunotoxins (ITs) are chimeric constructs obtained by linking a specific antibody to a toxin (e.g., proteins, peptides or other biological molecules with cytotoxic action) [1]. This strategy combines specific antibodies with cytotoxic molecules [2], in order to target the cytotoxic effect towards specific cell lines, killing them. Historically, Paul Ehrlich was the first to hypothesize the idea of a “magic bullet” (for disease treatment) endowed with a specific antigen not present in normal cells of the human body [3]. Nowadays, the possible use of monoclonal antibodies or antibody fragments, as well as the recombinant approach, improve the strategy for the obtainment of ITs [4]. Furthermore, some problems related to the use of ITs (e.g., off-target and on-target toxicities, immunogenicity, human cytotoxic proteins, antigen target selection and cytosolic delivery efficacy) that limit their clinical application can be overcome by new molecular approaches (e.g., affinity modulation, minimized immunoconstruct molecular weight, new drug delivery methods or nanomaterials) [5,6].

In this framework, several plant-derived (e.g., ribosome inactivating proteins (RIPs) [7]), fungi-derived (e.g., ribotoxins [8]), bacteria-derived (e.g., *Pseudomonas* exotoxin A [9] or diphtheria [10]) toxins are used for IT design and construction. Among them, many RIPs-based immunoconstructs are employed in preclinical and clinical studies [7,11].

RIPs are N-β-glycosylases (EC 3.2.2.22) able to remove a single adenine from the 28S rRNA (A_4324_, rat liver numbering), highly conserved in Sarcin Ricin Loop (SRL), and involved in ribosome interaction with prokaryotic or eukaryotic elongation factors (EF-G or EF-2, respectively) [12,13]. The SRL and, thus, ribosomes damaged by this enzymatic action are unable to perform protein synthesis [14], promoting cell death by apoptotic pathway [15]. RIPs are predominantly isolated from flowering plants (homologous proteins group [16,17]), although analogue enzymes able to inhibit protein synthesis are retrieved in fungi [12], bacteria [18] and one alga [19]. RIPs are classically divided into three groups due to the absence or presence of quaternary structure. Indeed, type 1 RIPs are basic and monomeric proteins (~30 kDa) with enzymatic action, while type 2 RIPs (~60 kDa) possess quaternary structure, being constituted by an A-chain (enzymatic action) and a B-chain (lectin function). On the other hand, type 3 RIPs contain both precursors (proRIPs) activated by proteolytic events [20] and active enzymes consisting of a type 1-like N-terminal domain (N-glycosylase domain) covalently linked to a C-terminal domain with unknown function [21]. The cytotoxic effect of these toxins depends on the different structural and enzymatic characteristics, although type 2 RIPs are usually more toxic than type 1 RIPs because lectin B-chain facilitates their internalization in cells, through membrane glycosylated receptor binding [22].

Research on RIPs is focused on their practical applications in medicine and agriculture. In medicine, RIPs are used to obtain ITs or other bioconjugates with the aim to eliminate malignant or specific target cells [11]. In agriculture, the potential use of RIPs exhibiting antiviral, antifungal and bioinsecticidal activities increases the resistance towards plant pathogens [23].

In this scenario, our research group recently isolated and characterized quinoin, a cytotoxic type 1 RIP from quinoa (*Chenopodium quinoa* Willd.) seeds [24], which possesses a low IC_50_ in vitro (5.08 pM; 0.15 ng/mL) [25] similar to saporin S6, a type 1 RIP isolated from *Saponaria officials* L. seeds (IC_50_ = 37 pM; 1.09 ng/mL), the latter widely used for immunoconjugate production [26]. Quinoin is a very stable (Tm = 68.2 °C) and basic protein of 254 amino acid residues, without cysteinyl residues [27]. In addition, this type 1 RIP possesses cytotoxic effects towards several human malignant cell lines by activating the apoptosis pathway [24,28] and exhibits antifungal activity against the green mould *Penicillium digitatum* [25]. Considering the above, quinoin is a promising candidate for possible use in the construction of novel ITs. Therefore, we decided to produce a novel IT by a chemical crosslinking approach, linking quinoin to cetuximab, a monoclonal antibody (mAb) blocking the epidermal growth factor receptor (EGFR).

Cetuximab is a therapeutic agent considering that EGFR is higher expressed in metastatic colorectal cancer (mCRC) and associated with cancer development and progression [29,30]. Cetuximab is an efficient substitute of chemical chemotherapeutic agents for patients with KRAS wild type mCRC [31]. On the other hand, evidence demonstrates that cetuximab treatment has no clinical benefits (progression-free survival and overall survival) in patients affected by mCRC with KRAS mutation [32,33] or in the presence of constitutive activation of signalling pathways acting downstream of the EGFR [33,34].

In this framework, the possible use of cetuximab-based ITs endowed with RIP toxicity and able to interact with EGFR receptors (overexpressed in mCRC cells) is a promising strategy, considering drug resistance. Thus, in this work we report the chemical strategy to obtain quinoin cetuximab immunoconjugate linked through SPDP and the enzymatic characterization of the obtained IT. Moreover, we evaluated the cytotoxic effect of this novel IT towards GEO-CR cell proliferation (cell line resistant to cetuximab) compared to the effect of cetuximab or quinoin alone. Indeed, it is known that GEO-CR cell line proliferation and survival signals are constitutively active despite EGFR inhibition by cetuximab treatment [33,34].

## 2. Results and Discussion

### 2.1. Quinoin Isolation

Quinoin was purified from the seeds of *C. quinoa* as previously reported [24]. The homogeneity of quinoin was achieved by both SDS-PAGE and RP-HPLC analysis (data not shown).

### 2.2. Chemical Linking between Cetuximab and Quinoin by Using SPDP

**Scheme 1:**
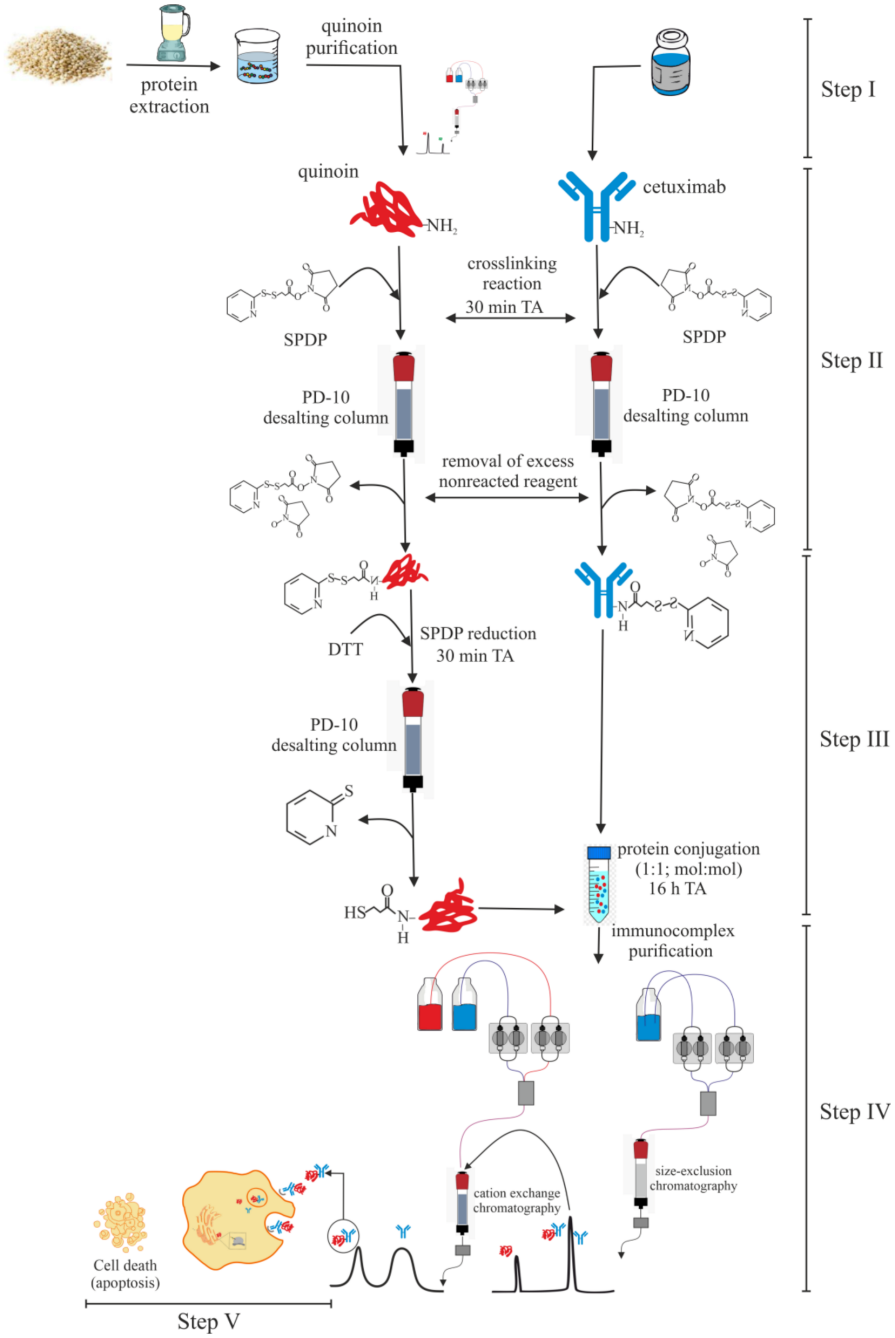
Flowchart of the experimental procedure used to obtain the quinoin cetuximab immunoconjugate. The following steps carried out: (I) toxin and antibody obtainment; (II) quinoin and cetuximab chemical modification; (III) chemical cross-linking; (IV) immunotoxin purification; and (V) cytotoxicity assays.

In order to obtain the specific IT with quinoin, cetuximab monoclonal antibody (Merck Serono S.p.A., Roma, Italy) was chemically linked to the type 1 RIP. Considering the absence of cysteinyl amino acid residues in the primary structure of quinoin [27], we chose SPDP as a heterobifunctional crosslinker to favour the formation of a disulphide bridge between cetuximab and the type 1 RIP by chemical modifications using the protocol described in the Section 4 [35] and in Scheme 1.

In particular, due to the absence of cysteinyl residues in the primary structure of quinoin, chemical conjugation was achieved by exploiting the reactivity of primary amines of this toxin (23 ε-amino group of lysinyl residues) [36]. Subsequently, considering the presence of disulphide bridges in cetuximab structure, we decided to reduce quinoin in order to obtain the final conjugate (steps II and III, Scheme 1).

**Figure 1:**
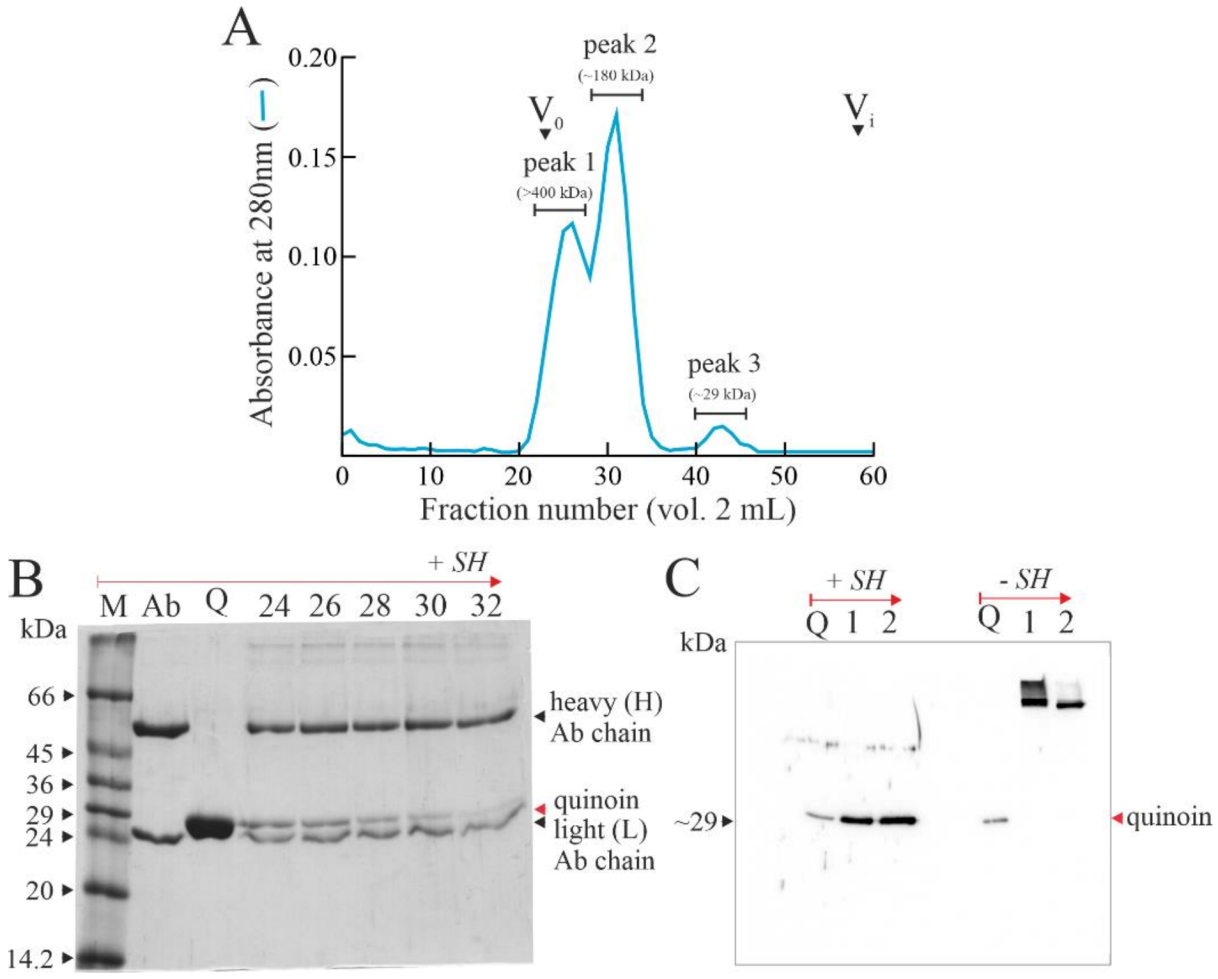
Purification of immunocomplex mixture after SPDP reaction. (**A**) Chromatographic profile from gel-filtration on Superdex 200 column of cetuximab and quinoin after SPDP reaction performed as described in the Section 4. V_0_ and V_i_ indicate void and inclusion volume of gel-filtration column, respectively. (**B**) SDS-PAGE on a 12% polyacrylamide gel under reducing conditions (+SH). M, molecular weight markers; Ab and Q, cetuximab and quinoin, respectively; lanes 24–32, peak fractions from gel-filtration. (**C**) Western blotting of peak 1 and 2 (~200 ng) from gel filtration after SDS-PAGE with and without reducing agent, +SH and −SH, respectively, using anti-quinoin polyclonal rabbit antibody.

In the last step of quinoin cetuximab immunoconjugate purification, to remove unreacted SPDP-modified quinoin, this reaction mixture was subjected to gel filtration on the Superdex^®^ 200. As shown in Figure 1A, three protein peaks, named peak 1, 2 and 3 with a molecular weight of >400, ~180 and ~29 kDa, respectively, were eluted. In order to verify the fractions in which quinoin is present, the polynucleotide: adenosine glycosylase (PNAG) assay was carried out (data not shown). Peak 3 fractions, corresponding to the elution volume of native quinoin, were endowed with PNAG activity; the same activity was detected also in peak 1 and 2 fractions, eluted at lower elution volume corresponding to higher molecular weight proteins (>150 kDa), suggesting that quinoin was linked to the antibody.

The fractions corresponding to peak 1 and 2 were analysed by SDS-PAGE in the presence of reducing agent (Figure 1B). Each fraction revealed the presence of a protein band with an electrophoretic migration of ~29 kDa, corresponding to quinoin released from the immunoconjugate, following the breaking of chemical disulphide bridge under reducing conditions. Thus, peaks 1 and 2 fractions were pooled and analysed by Western blot with or without reducing agent to verify the covalent bond between cetuximab and quinoin (Figure 1C). The analysis of peak 1 and 2 showed that in the absence of reducing agent, the cross-reactive bands were evident at higher molecular weight, while under reducing conditions, a single cross-reactive band with an electrophoretic migration of ~29 kDa appeared, confirming the linking through chemical disulphide bridge between cetuximab and quinoin.

On the other hand, Western blot analysis without reducing agent showed that peak 1 corresponded to a heterogeneous conjugation due to the presence of more cross-reactive bands, while peak 2 (~180 kDa) had a single cross-reactive band. Thus, considering the molecular weight of the single cross-reactive band and the PNGA activity of peak 2, it is possible to affirm that peak 2 corresponded to quinoin cetuximab immunoconjugate, whereas peak 1 likely consisted of immunoconjugate heterogenous forms in which the ratio quinoin cetuximab was higher than 1:1, justifying the higher molecular weight retrieved.

**Figure 2:**
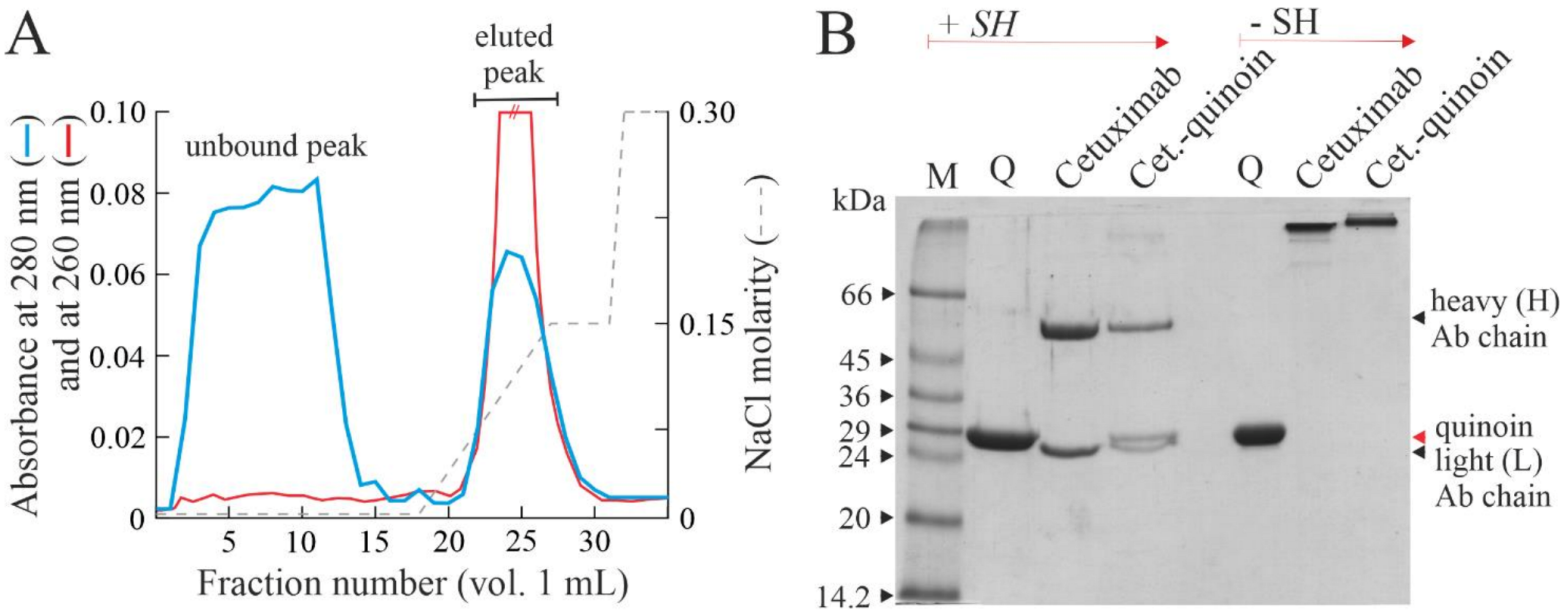
Purification of cetuximab quinoin immunocomplex (Cet.-quinoin). (**A**) Elution profile from cation exchange chromatography on a Source 15S column of immunocomplex pool (peak 2 in Figure 1A) and PNAG activity profile reported as absorption increase at 260 nm (see Section 4). (**B**) SDS-PAGE on a 12% polyacrylamide gel of Cet.-quinoin eluted pool (eluted peak in A) with (+SH) or without (−SH) reducing agent. Lanes M and Q, molecular markers and quinoin, respectively; lanes cetuximab and Cet.-quinoin (3.0 µg), antibody (3.0 µg) and immunocomplex (3.0 µg), respectively.

Furthermore, to improve the purity of peak 2, we included a further purification step based on cation exchange chromatography (Figure 2A). Two main protein peaks were eluted, an unbound peak without PNAG activity, containing the unconjugated antibody, and an eluted peak with PNAG activity (Figure 2A).

The active fractions of eluted peaks were pooled and analysed by SDS-PAGE to verify the homogeneity of quinoin cetuximab immunoconjugate, hereafter named Cet.-quinoin (Figure 2B). Under reducing conditions, three protein bands with an electrophoretic migration of 50 kDa, 29 kDa and 25 kDa corresponding to heavy antibody chain, quinoin and light antibody chain were respectively detected, while under non-reducing conditions, a single protein band with an electrophoretic migration of ~180 kDa slightly higher than cetuximab alone (~150 kDa) was observed, confirming the immunoconjugate homogeneity.

Typically, starting from 2.4 mg (80 nmol) of quinoin and 12 mg (80 nmol) of cetuximab, the setup strategy allowed us to obtain 0.5 mg of Cet.-quinoin (Scheme 1, steps II–IV). The Cet.-quinoin concentration typically obtained after the chemical conjugation and purification was about 0.36 mg/mL.

### 2.3. Enzymatic Properties of Cetuximab Quinoin Immunocomplex

**Figure 3:**
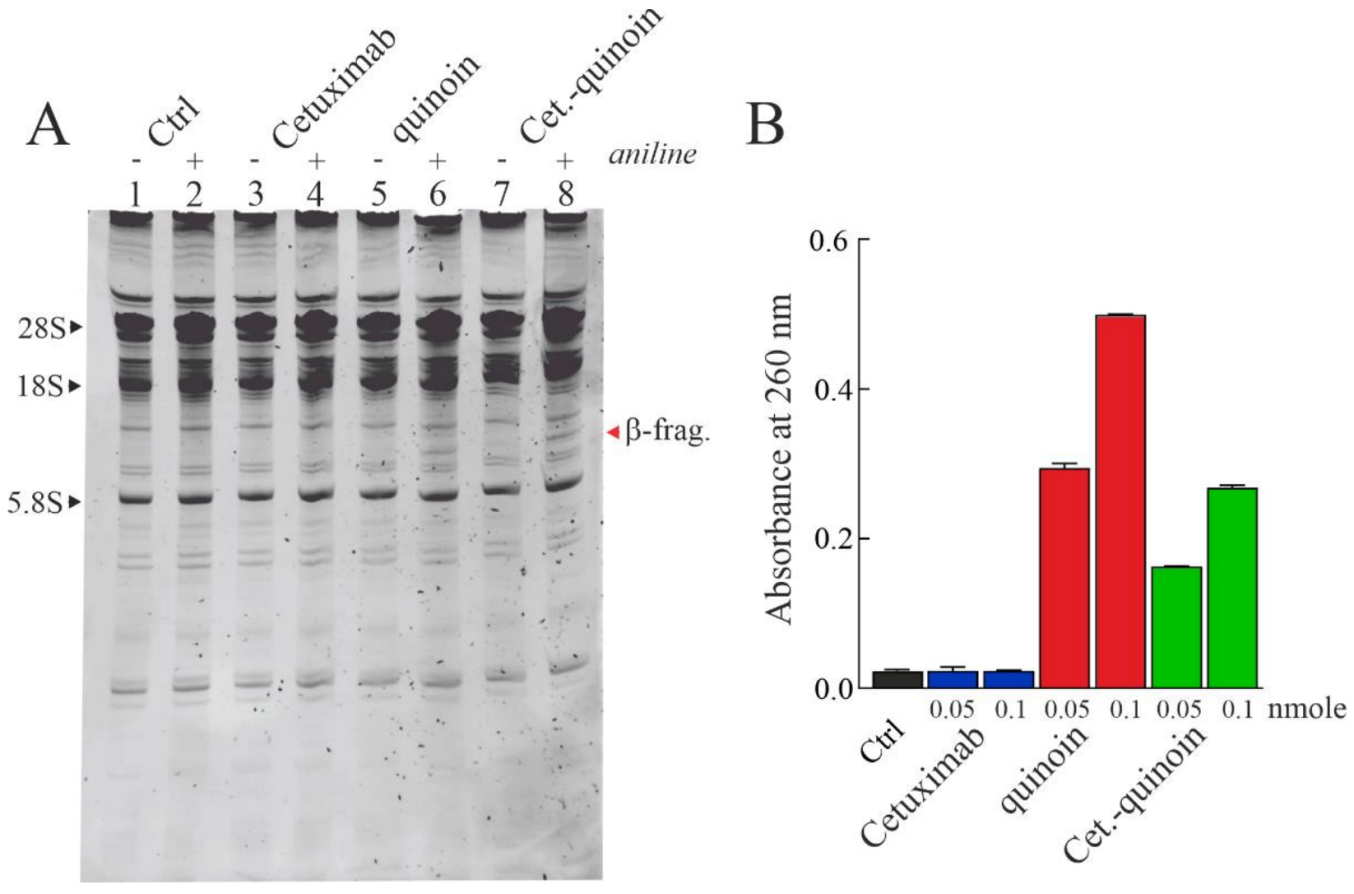
Enzymatic activity of quinoin cetuximab immunoconjugate. (**A**) rRNA N-β-glycosylase activity on rabbit ribosomes. Ctrl, untreated control (3.0 µg; lanes 1 and 2), Cetuximab (3.0 µg; lanes 3 and 4) as a negative control and quinoin (3.0 µg; lanes 5 and 6) as a positive control. Lanes 7 and 8, quinoin cetuximab immunoconjugate (Cet.-quinoin, 3.0 µg), were incubated with rabbit ribosomes. Then, rRNA was extracted, treated with acid aniline and separated as described in the Section 4. (+) and (−) indicate with and without aniline treatment. “β-frag” indicates the position of Endo’s fragment released by aniline treatment of rRNA from rabbit ribosomes. (**B**) Polynucleotide:adenosine glycosylase (PNAG) activity of cetuximab (negative control), quinoin and quinoin cetuximab immunoconjugate (Cet.-quinoin). Ctrl, untreated control. Equimolar quantity of proteins (0.05 and 0.10 nmole) was assayed on salmon sperm DNA as described in the Section 4. The mean results ± SD of three experiments performed in triplicate are reported.

N-β-glycosylase activity of RIPs such as quinoin consists in hydrolysing the N-β-glycosidic bond of a specific adenine in the SRL region of 28S rRNA (for further details, see Section 1 and [37]). The consequent formation of an apurinic site prevents the interaction between the elongation factors (EF-G or EF-2 in prokaryotes and eukaryotes, respectively) and the ribosomes, blocking translocation and inhibiting protein synthesis, triggering the apoptotic pathway. In this framework, Endo’s assay is considered a valuable method to check N-β-glycosylase activity, typical of RIPs. Thus, in order to confirm that Cet.-quinoin is able to deadenylate ribosomes, cetuximab, native quinoin and Cet.-quinoin were assayed on rabbit ribosomes. As shown in Figure 3A, Cet.-quinoin caused the release of diagnostic β-fragment, like quinoin, confirming that N-β-glycosylase activity was retained after chemical linking.

On the other hand, PNAG activity of Cet.-quinoin decreased ~36% relative to native quinoin when equimolar quantities were assayed (Figure 3B). This finding is likely associated with an acquired impairment of adenine recognition by quinoin linked to the antibody. Probably, different interactions are necessary between quinoin and native ribosome compared to a heterogeneous substrate such as salmon sperm DNA considering the high molecular weight of immunoconjugate relative to quinoin alone [38].

### 2.4. In Vitro Effects of Quinoin Cetuximab Immunoconjugate on Cell Proliferation and Apoptosis Using Model Colon Cancer Cells with Acquired Resistance to Cetuximab

**Figure 4:**
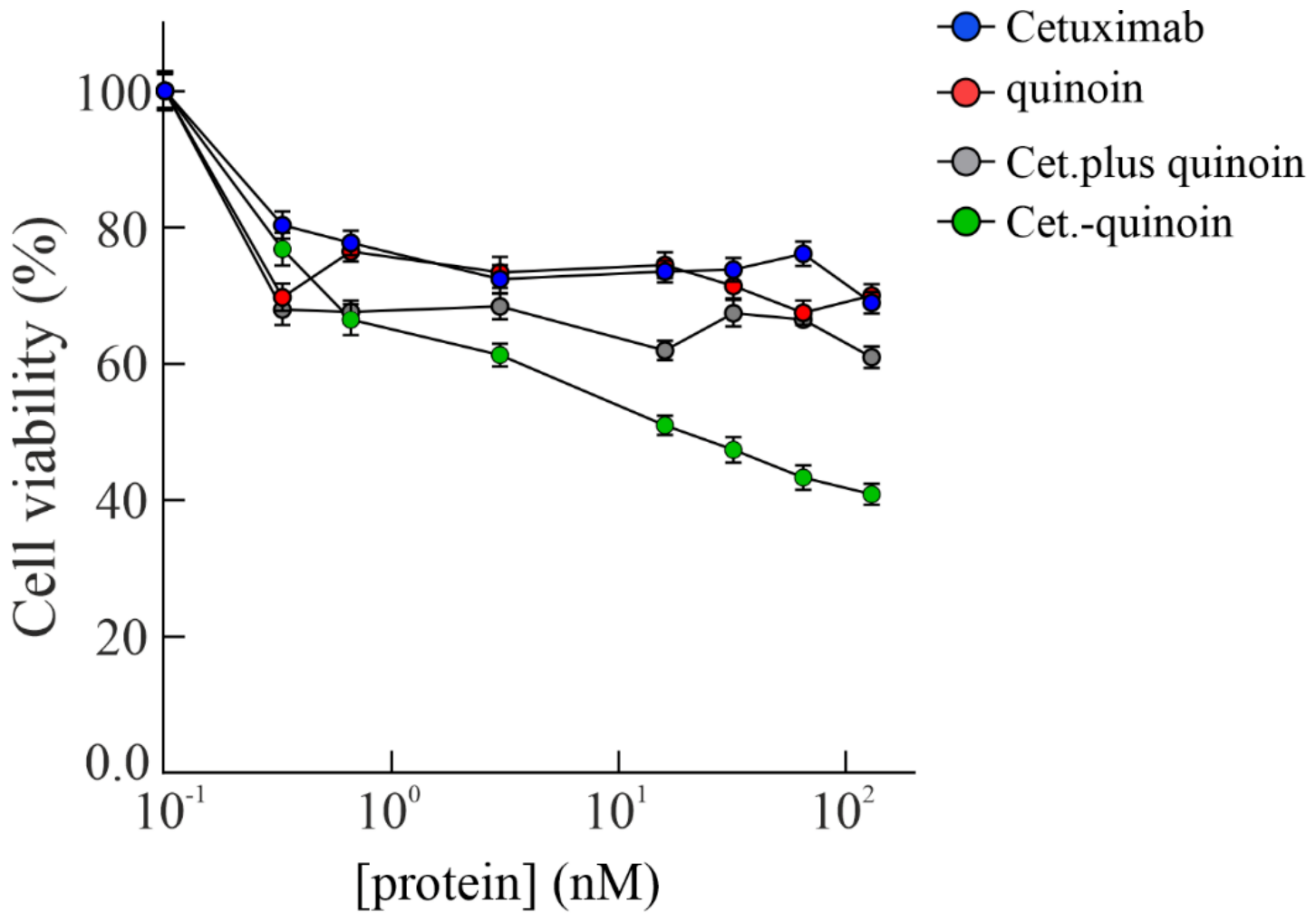
Effect of cetuximab, quinoin, quinoin cetuximab immunoconjugate (Cet.-quinoin) and the combination of cetuximab plus quinoin (Cet. plus quinoin) on cetuximab-resistant GEO cancer cell line (GEO-CR) viability. Cells were treated with different concentrations of drugs (range of doses: from 0.33 to 130 nM) for 72 h. The viability was determined using the MTT assay. Every point is the mean ± SD of three independent experiments with three repetitions.

The sensitivity of GEO-CR cell lines was evaluated through a cell proliferation assay in the presence of increasing doses of cetuximab, quinoin, Cet.-quinoin or the combination of cetuximab plus quinoin (Cet. plus quinoin) in a dose ranging from 0.33 to 130 nM, considering a molecular weight of ~29, ~150 and ~180 kDa for quinoin, cetuximab and Cet.-quinoin, respectively (Figure 4). A progressive decrease in cell proliferation in a dose-dependent manner was observed after 72 h. In particular, cells behaved with similar sensitivity to cetuximab, quinoin and their combination, maintaining 60–70% of cells surviving with the higher doses of 130 nM. In contrast, treatment with Cet.-quinoin resulted in a significant effect on cell viability. Indeed, the higher concentration of Cet.-quinoin reduced GEO-CR cell growth by more than 60% (40% of cell viability) with the highest dose (130 nM) at 72 h, reaching an IC_50_ value at the dose of 27.7 nM (~5.0 μg/mL), while in the same conditions, the IC_50_ values of cetuximab and quinoin were 176.7 and 149.3 nM, respectively. This evidence demonstrates the efficacy of the immunoconjugate on GEO-CR cells compared to the single drugs. Therefore, in this condition, the fold cytotoxic potentiation of the conjugate highest dose at 72 h was 1.90, 1.97 and 1.51 compared to cetuximab, quinoin or the combination of cetuximab plus quinoin, respectively.

Finally, since the increase in anti-proliferative effects is generally associated with an increase in apoptotic rate, the same experiment was conducted by treating GEO-CR cells with a single dose corresponding to the IC_50_ value (27.7 nM) for 48 h to evaluate the ability of drugs to induce apoptosis using the Annexin V-FITC assay.

**Figure 5:**
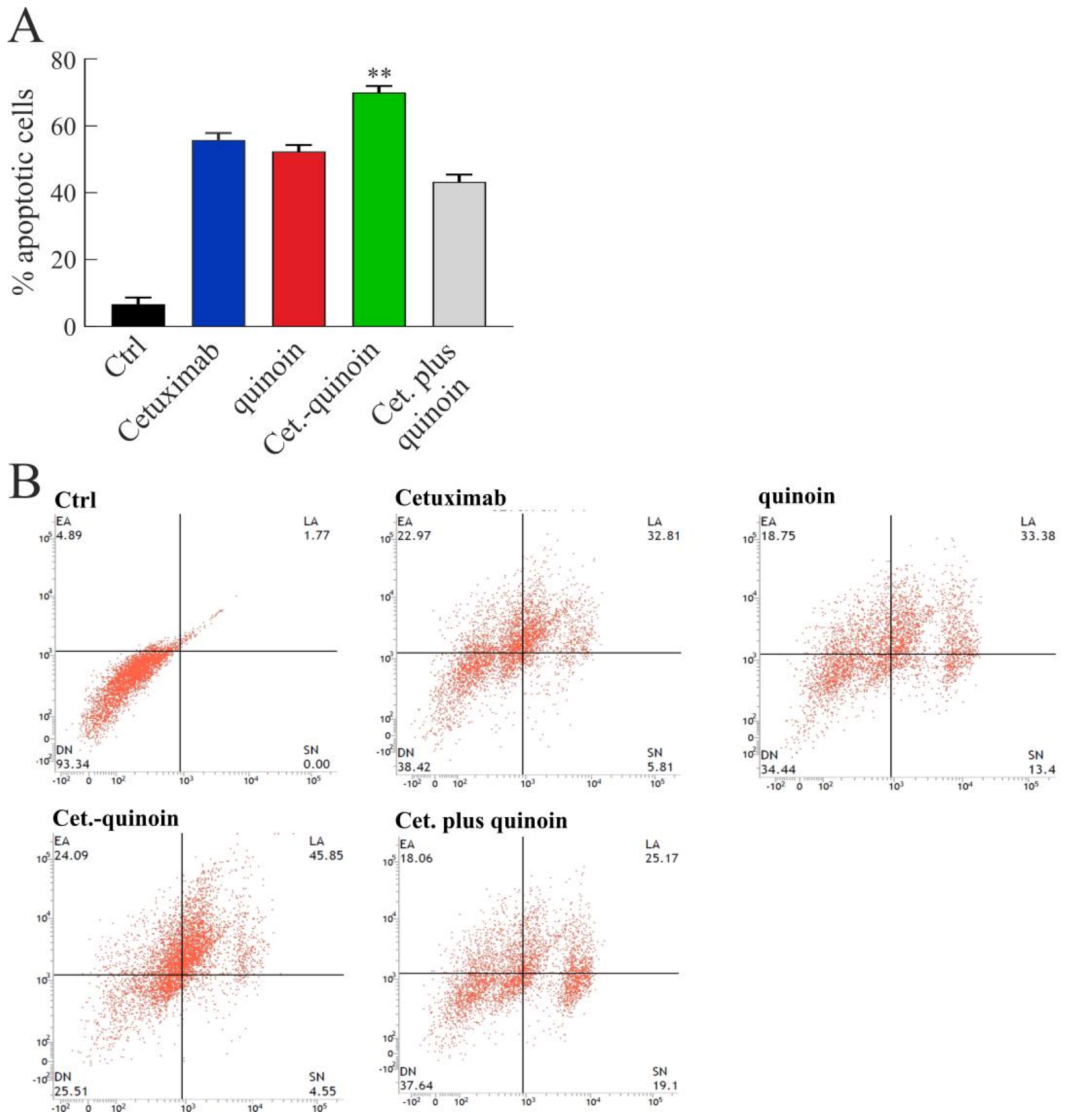
Effect of cetuximab, quinoin, quinoin cetuximab immunoconjugate (Cet.-quinoin) or the combination of cetuximab plus quinoin (Cet. plus quinoin) treatment on cell viability in cetuximab-resistant GEO cancer cell line (GEO-CR). Ctrl indicates the untreated control. (**A**) Histogram of data expressed as percentage of apoptotic cells. Bars represent mean values obtained from three separate experiments. *p* values < 0.05 were considered as statistically significant (**). (**B**) Representative flow cytometric analysis of GEO-CR cell apoptosis. One representative experiment is shown. Dot plot diagrams show the different stages of apoptosis: % indicated in the upper left quadrant represents cells positive for annexin V and negative for PI, considered as early apoptotic cells; % in upper right quadrant indicate cells positive for both annexin V and PI, showing the late apoptotic cell population; % in lower left quadrant represents viable cells, negative for both markers, and % in lower right quadrant indicates cells negative for annexin V or necrotic cells.

As shown in Figure 5, no apoptotic events were observed in the control cells, while after a single treatment with cetuximab, quinoin or cetuximab plus quinoin, an apoptotic rate of 55%, 52% and 43%, respectively, was observed. However, in the same experimental condition, the treatment with Cet.-quinoin reached an apoptotic rate of 70%, demonstrating the strong effect of this complex to induce early and late stages of apoptosis and consequently cell death.

Overall, quinoin delivery into the cell is mandatory for the improved cytotoxic action of this IT, as reported for other ITs, and it may be that Cet.-quinoin follows a mechanism based on: (i) IT-EGFR binding; (ii) IT-EGFR complex internalization; (iii) quinoin release from IT by disulphide bridge breaking; and (iv) enzymatic action of quinoin on ribosomes after reaching the cytosol [39,40,41]. However, since the precise mechanism of said complex pathway is not fully understood, further research is required [42].

## 3. Conclusions

In this work, a novel immunotoxin linking cetuximab to quinoin, type 1 RIP from quinoa seeds, was obtained using heterobifunctional reagent SPDP. Cet.-quinoin retains the enzymatic activities of native quinoin, showing increased cytotoxicity towards GEO-CR cells (cell line resistant to cetuximab) compared to cetuximab or quinoin alone. Moreover, cytofluorimetric analysis suggested that Cet.-quinoin improves cell death through the apoptotic pathway compared to cetuximab or quinoin alone, as well as quinoin plus cetuximab.

Overall, although this novel immunoconjugate against cell lines resistant to cetuximab does not exhibit the desired selective cytotoxic effect, the set-up protocol could be useful for the development of novel immunoconjugates able to increase the clinical benefits for patients with a lower EGFR expression.

## 4. Materials and Methods

### 4.1. Materials

Materials for chromatography were described elsewhere [24,43,44]. All other reagents and chemicals (e.g., single-stranded salmon sperm DNA, dithiothreitol (DTT) and urea) were of analytical grade (Merck Life Sciences S.r.l., Milano, Italy). The nuclease-treated rabbit reticulocyte lysate system was purchased from Promega (Madison, WI, USA). Cetuximab, an anti-EGFR human-mouse chimeric mAb, was provided by Merck Serono S.p.A. (Rome, Italy).

### 4.2. Quinoin, Type 1 RIP Purification

Quinoin was purified from quinoa seeds following the procedure previously reported [24]. Quinoin purity and integrity were checked by SDS-PAGE analysis. Then, the protein was dialysed against deionised water and concentrated (~5 mg/mL). Finally, aliquots were transferred into 1.0 mL sample polypropylene vials and stored at −20 °C until use.

### 4.3. Immunoconjugate Preparation

Quinoin was conjugated to cetuximab monoclonal antibody using the heterobifunctional reagent succinimidyl 3-(2-pyridyldithio)propionate (SPDP; Thermo Fisher Scientific Inc. Rodano, Milano, Italy) [35,45]. Modified quinoin and cetuximab were obtained by reaction with a molar excess of SPDP of 5 and 20, respectively in phosphate buffered saline buffer with EDTA (PBS-EDTA; 100 mM Na-phosphate, 150 mM NaCl, 1.0 mM EDTA, pH 7.5). After 30 min of incubation, the excess of SPDP was removed by gel filtration chromatography on PD-10-Cytiva desalting column (Merck Life Science S.r.l.) with PBS-EDTA. Subsequently, the pyridyl disulphide-quinoin was treated whit an excess of 50 mM DTT for 30 min at room temperature. The excess of DTT was removed by gel filtration on PD-10-Cytiva column with PBS-EDTA. Then, the reduced quinoin was mixed with the pyridyl disulphide-cetuximab at a 1:1 molar ratio for 16 h, at 25 °C. Subsequently, the immunoconjugate mixture was gel-filtrated by FPLC on an AKTA Purifier System (Cytiva, Buccinasco (MI), Italy) using a HiLoad^®^ 16/60 Superdex^®^ column (L × I.D. 60 cm × 16 mm; range 600–10 kDa; Cytiva, Buccinasco (MI), Italy), equilibrated and eluted in PBS without EDTA, pH 7.5 (flow rate 1.0 mL/min), and monitored for the absorbance at 280 nm.

### 4.4. Immunoconjugate Purification

Cetuximab quinoin immunoconjugate mixture (~180 kDa) after gel filtration was further subjected to cation exchange chromatography on an AKTA Purifier System (Cytiva) using a Source 15S PE 4.6/100 column (vol. 1.6 mL, Cytiva), equilibrated in 5.0 mM Na-phosphate, pH 7.2 and eluted in the same buffer containing 0.3 M NaCl (buffer B), applying a discontinuous gradient (total time: 40 min.) at a flow-rate of 1.0 mL/min. After loading samples, discontinuous gradient steps were: (i) isocratic elution with buffer A for 17 min; (ii) increasing concentration of buffer B to 50% (0.15 M) in 11 min; (iii) increasing concentration of buffer B to 100% (0.30 M) in 2 min; (iv) washing with 100% of buffer B for 25 min.

### 4.5. Analytical Procedures

Quinoin or immunoconjugate purity and integrity were determined by SDS-PAGE with a Mini-Protean II mini-gel apparatus (Bio-Rad, Rome, Italy) using 6% stacking and 15% separation polyacrylamide gel; precision plus protein kit (Bio-Rad, Hercules, CA, USA) was used for reference proteins. Protein concentration was determined by Pierce BCA Protein Assay kit (Thermo Fisher Scientific, Rodano, Italy), using BSA as standard. Western blot analyses were performed after SDS-PAGE separation, transferring proteins onto nitrocellulose membrane (filter type 0.45 µm HATF; Millipore, Burlington, MA, USA) by electroblotting with Mini Trans-Blot Cell (Bio-Rad, Hercules, CA, USA). Finally, the blot was probed with anti-quinoin polyclonal rabbit antibody (Bio-Fab research, Rome, Italy) as a primary antibody (dilution 1:2500). Immunoreactive bands were detected following incubation with the anti-rabbit HRP-conjugated antibody (Bio-Rad, 1:3000), by adding the Clarity^TM^. Western ECL substrate (Bio-Rad, Hercules, CA, USA) and acquired by using the ChemiDoc XRS System (Bio-Rad, Hercules, CA, USA).

### 4.6. Enzymatic Assays

*rRNA N-β-glycosylase assay*. Depurination assay to detect the β-fragment (Endo’s fragment) was conducted as described by [37,46]. Rabbit reticulocyte lysate was used as substrate for both quinoin and immunoconstruct. Briefly, after rabbit ribosome (80 µL) incubation with proteins (3.0 µg), rRNA was extracted by phenolization, treated with 1 M aniline acetate (pH 4.5) and precipitated with ethanol. rRNA was subjected to electrophoresis at 15 mA in a 7 M urea/5% (*w*/*v*) polyacrylamide gel for a suitable time and stained with ethidium bromide.

*PNAG on salmon sperm DNA assay*. Adenine release was measured according to the method reported by [47]. Ten micrograms of salmon sperm DNA (substrate) were incubated with 0.05 or 0.1 nmole of quinoin (1.5 or 3.0 μg) or immunoconjugate (9.0 or 18 μg) in 300 μL of a reaction buffer, pH 4.0, at 30 °C for 60 min. After incubation, DNA was precipitated with ethanol at −80 °C (overnight) and centrifugated at 18,000 g (15 min) at 4 °C. Adenine release was determined in the supernatants with a Cary 50 UV-Vis spectrophotometer (Agilent Technologies Italia S.p.A., Cernusco sul Naviglio (MI), Italy) at 260 nm.

### 4.7. Cell Lines and Drugs

Human colon cancer GEO cell line was provided by American Type Culture Collection (ATCC, Manassas, VA, USA) and maintained in Dulbecco’s Modified Eagle Medium (Merck Life Science S.r.l.) supplemented with 20% FBS (FBS; Life Technologies, Gaithersburg, MD, USA), 1% penicillin/streptomycin (Merck Life Science S.r.l.) in a humidified atmosphere with 5% CO_2_ at 37 °C. The identity of the cell line was confirmed by Human Cell STR Testing (ATCC) on an ad hoc basis prior to performing experiments and repeated after the majority of the experiments were performed. For a period of 6 months, GEO cells were continuously exposed to increasing concentrations of cetuximab (Merck Serono, Rome, Italy), starting from the dose causing the 50% inhibition of cancer cell growth (IC_50_). The established cetuximab-resistant GEO cancer cell line (GEO-CR) was then maintained in continuous culture with the maximally achieved dose of cetuximab allowing cellular proliferation [33,34]. All cell lines were routinely screened for the presence of mycoplasma (Mycoplasma Detection Kit, Roche Diagnostics, Monza, Italy).

### 4.8. Cell Viability Assay

Cells were seeded in 24-well plates at the density of 1 × 10^4^ cells/well and treated with increasing doses of drugs from 0.33 to 130 nM (i.e., 0.33, 0.66, 3.0, 16, 32, 65 and 130 nM) for 72 h. Cell proliferation was measured with 3-(4,5-dimethylthiazol-2-yl)-2,5-diphenyltetrazoliumbromide (MTT, Merck Life Science S.r.l.). The concentrations inhibiting 50% of cell growth (IC_50_), determined by interpolation from the dose–response curve and the corresponding values, were used for subsequent experiments. Results represent the mean of three separate experiments, each performed in quadruplicate.

### 4.9. Apoptosis Assessment

Cells were cultured in a 6-well plate (1 × 10^4^ cells/well) and treated with 27.7 nM of each drug. After 48 h treatments, apoptosis was evaluated by flow cytometry using AnnexinV-FITC and Propidium iodide (PI) double staining (Life Technologies Italia Fil., Monza, Italy) according to the manufacturer’s instructions. The detection of viable cells, early and late apoptotic cells, and necrotic cells was performed by BD Accuri™ C6 (BD Biosciences, Milan, Italy) flow cytometer and subsequently analysed by ACCURI C6 software version 264.21 (BD Biosciences). Results represent the mean of three separate experiments, each performed in duplicate.

### 4.10. Statistical Analysis

Statistical analysis of in vitro data was performed using a one-way analysis of variance (ANOVA). Quantitative data were reported as mean ± standard deviation (SD) from three or more independent experiments. Results were compared by analysis of variance (ANOVA) followed by the Student t-test. Data were processed by using Prism GraphPad (GraphPad Software, San Diego, CA, USA).

## Data Availability

The data presented in this study are available in this article.

## Abbreviations

PNAG: polynucleotide:adenosine glycosylase; EGFR, epidermal growth factor receptor; mCRC, metastatic colorectal cancer; RIPs, ribosome inactivating proteins; SRL, sarcin ricin loop.
